# Quantitative evaluation and optimization of AI policy and regulatory texts for smart healthcare

**DOI:** 10.3389/fpubh.2025.1731013

**Published:** 2025-12-12

**Authors:** Zhaolin Zhou, Yu Xiang, Chunchun Liu, Xinru Huang, Yan Fu

**Affiliations:** 1 School of Management, Xuzhou Medical University, Xuzhou, China; 2 Research Institute Chinese-Style Modernization in Healthcare, Xuzhou Medical University, Xuzhou, Jiangsu, China

**Keywords:** smart healthcare, policy quantification, policy tools, content analysis method, PMC-Index model

## Abstract

**Background:**

Artificial intelligence has revolutionized the field of smart healthcare, demonstrating significant value in enhancing diagnostic and treatment efficiency and controlling medical costs. AI in smart healthcare system policies are of great significance for optimizing the allocation of medical resources, promoting the accuracy and efficiency of diagnosis and treatment services. Evaluation of AI in smart healthcare system policy texts can provide theoretical support and decision-making basis for the scientific formulation, effective implementation, adjustment and optimization of AI in smart healthcare system policies.

**Methods:**

The study analyzes 10 representative policy texts from 77 policies during 2015–2025, and the strengths and weaknesses of each policy and the optimization and adjustment paths are analyzed by calculating the PMC index and drawing PMC surface and radar diagrams.

**Results:**

The findings reveal that the overall quality of AI policies for smart healthcare reaches an “excellent” level, with notable strengths in policy focus and the completeness of evaluation systems. However, challenges persist, including insufficient policy continuity, overreliance on mandatory directives as policy tools, and weak operability of policy measures.

**Conclusion:**

The study utilizes the PMC policy standardization assessment to identify policy issues and provides differentiated design references based on regional differences, offering crucial support for the collaborative improvement, scientific construction, and global AI governance optimization of the international AI policy framework.

## 1 Introduction

The World Health Organization released its New Guidance on Multimodal Large Model Governance. The document outlines application scenarios for multimodal large models in healthcare, including pathological diagnosis, patient self-management, administrative tasks, medical education and research (1). In recent years, the depth and breadth of artificial intelligence applications in smart healthcare have continued to expand. AI is viewed as an opportunity to advance medical progress by facilitating the storage, analysis, and interpretation of vast amounts of data, while enhancing diagnostic accuracy and speed and optimizing treatment strategies (2). Additionally, from the overview of 10 OECD countries, AI-based systems have the potential to improve medical healthcare by preventing hospitalizations, reducing administrative burdens, and improving patient engagement (3). However, this has also created governance challenges concerning ethical implications (4), data security (5, 6), and equitable access (7). Smart healthcare policies constitute the institutional frameworks and top-level designs through which various departments drive the development of the smart healthcare industry. The quality of these policy texts directly impacts not only the quality and efficiency of smart healthcare services but also the effectiveness and future prospects of AI technology applications in the medical field. Rush et al. (8) adopted the qualitative content analysis for the audit of AI-related documents across U.S. Medical schools. Gu et al. (9) applied word frequency analysis to study smart service policies in China’s healthcare sector, with limited exploration of quantitative method. Research on the relationship between the AI in smart healthcare and quantify policy text is relatively scarce. To address this gap, this paper adopts the smart healthcare perspective and employs the PMC-Index model to scientifically evaluate the formulation effectiveness of various smart healthcare policies. It compares policy differences, analyzes current characteristics and focal points of smart healthcare policies, deconstructs and assesses their implementation effectiveness, and proposes targeted optimization recommendations and pathways. These efforts aim to advance smart healthcare development, strengthen governance capabilities and outcomes regarding AI-related social risks, and provide quantitative evidence to inform policy in AI in smart healthcare. To facilitate analysis, this paper poses the following three questions: (1) What are the key factors influencing the effective implementation of AI in smart healthcare policies and regulations? (2) What methods can be employed to assess and analyze the governance efficiency of AI in smart healthcare policies and regulations? (3) What pathways exist to enhance the governance efficiency of AI in smart healthcare policies and regulations, and how can they be implemented?

## 2 Literature review

### 2.1 Governance research on AI in smart healthcare

Artificial intelligence is reshaping the development paradigm of healthcare with transformative force (10). From AI-assisted interpretation of nuclear medicine in oncology and image generation services (11), to clinical AI readiness assessors enhancing diagnostic accuracy and operational efficiency while improving patient care (12), to the growing prominence of AI in veterinary medicine where numerous commercial veterinary AI products have already entered the market (13). The value of technology-enabled advancements is already evident in scenarios such as enhanced diagnostic and treatment efficiency (14) and healthcare cost control (15). The World Health Organization explicitly states in its Global Strategy on Digital Health (2020–2025) that AI serves as the core driver accelerating informatization upgrade in healthcare, with its development and regulation emerging as critical issues in global health governance. However, the governance lag risk underlying this technological leap forward must not be overlooked. AI in smart healthcare inherently involves data sensitivity, irreversible outcomes, and complex liability. Its governance extends far beyond mere technical regulation, constituting a multidimensional systemic endeavor encompassing legal, ethical, and societal dimensions. Against this backdrop, this paper analyzes core contradictions and explores implementation pathways for generative AI in smart healthcare across three dimensions: technological advancement, risk prevention and control, and value realization.

#### 2.1.1 Research on technical risks of AI in smart healthcare

The technical characteristics of artificial intelligence determine the complexity of its governance in the healthcare sector. For instance, Jia and Zhao (16) proposed from a global governance perspective that as generative AI becomes more deeply embedded in healthcare, enhanced global dialogue and cooperation are essential to ensure technological safety. Sosna et al. (17) focused on technical challenges in generative AI healthcare, such as data availability, and advocate for sustained multidisciplinary efforts to enhance AI safety. Kiseleva et al. (18) suggested addressing AI’s “black box problem” by treating transparency as a multi-layered system. This involves balancing algorithmic opacity through enhanced transparency measures from AI providers and other relevant stakeholders. Zhang and Zou (19) pointed out that when adopting generative AI technologies for myopia treatment, establishing multi-center, well-labeled datasets and conducting repeated tests using multiple state-of-the-art algorithms on the same task can establish benchmarks for algorithm performance and provide references for further developing relevant evaluation systems. In summary, while scholars have explored the technical risks of generative AI in healthcare from multiple dimensions, policy and regulatory frameworks lag behind the rapid advancement of science and technology. This creates numerous potential hazards in the application of generative AI within medical settings and undermines efforts to enhance social governance efficiency.

#### 2.1.2 Ethical research in AI in smart healthcare

Addressing ethical concerns in AI-generated content, Stewart McLean’s team (20) proposed an “embedded ethics” approach. In this iterative and continuous process, ethicists and developers collaborate from the outset to resolve ethical issues, potentially serving as an effective means to integrate robust ethical considerations into the practical development of AI in smart healthcare. Zhang (21) advocated prioritizing ethical values that promote human health as a top-level design principle, while intensifying risk assessment and regulatory oversight. Katznelson and Gerke (22) emphasized the necessity of teaching health AI ethics in medical schools to foster an ethical understanding of its ongoing development and equip professionals to address emerging ethical challenges posed by health AI or other digital health technologies. Goisauf and Cano (23) conducted a systematic review of academic literature, revealing that certain ethical and social impacts of AI usage remain under-explored. They call for greater attention to addressing potential discriminatory effects and injustices. Mohammed et al. (24) emphasized the importance of understanding data owner rights and establishing ethical norms for AI use in healthcare applications. Their research makes valuable contributions to the AI ethics debate and assists nursing and healthcare professionals in developing ethical AI practices. Jensen et al. (25) primarily focuses on the ethical implications of AI in smart healthcare and proposed “embedded ethics” approach. Specifically, embedded ethics provides multi-dimensional information support for policy design. For one, it exposes potential ethical risks in the initial stages of AI development, providing a basis for policy formulation to avoid post-facto remediation. For another, ethical review standards developed in practice can be directly translated into mandatory policy indicators, transforming principled advocacy into actionable rules and enhancing regulatory precision. While scholars have extensively researched the ethical challenges of AI in smart healthcare, further research and practical exploration are needed to balance data owner rights with the demands of AI in smart healthcare development across different regions and healthcare settings, and to ensure the effective implementation of ethical norms.

#### 2.1.3 Practical research on AI in smart healthcare

AI in anesthesiology can enhance diagnostic and therapeutic efficiency, and ultimately reduce healthcare costs (26). Furthermore, AI is applied to robot-assisted thoracic surgery and perioperative management to improve surgical accuracy (27). Simultaneously, artificial intelligence will fundamentally transform the workflow of modern dental clinics. Automated cone-beam computed tomography segmentation can help professionals obtain accurate 3D images in less time, thereby enhancing the efficiency of the entire process (28). Despite the widespread application of artificial intelligence in various medical specialties globally, there are significant differences in deployment priorities and focus areas among countries. The Da Vinci surgical robot, developed in the United States, is primarily applied in specialized clinical fields such as urology, gynecology, and cardiac surgery. In contrast, Germany, Japan, and other countries facing severe aging demographics have widely integrated AI into older adults care models (29), with a focus on chronic disease management, remote monitoring, and long-term care support. This divergence stems from differences in national healthcare priorities: the U.S. emphasizes advanced clinical technology adoption driven by medical specialization, while aging societies like Germany and Japan prioritize AI applications that address the unmet needs of older population. Such comparisons highlight how the goals of health policy in different countries collectively shape the direction of smart healthcare. In summary, the practical application of AI in various medical scenarios not only demonstrates its significant medical value but also reflects the potential of technological innovation. The differences in medical needs and development priorities among countries provide the key practical basis for the formulation of healthcare AI policies, and thus promote countries to formulate highly adaptable special policies based on their own medical resource endowment, core needs and development goals.

Overall, artificial intelligence in smart healthcare has become a major global concern, with academia beginning to focus on the field of smart healthcare policy research. However, evaluation studies targeting the policy texts themselves remain relatively scarce. To address this gap, this research introduces the PMC index model into the field of smart healthcare policy studies, providing a reference framework for policy formulation. The ultimate objective of this paper is to conduct quantitative evaluation and optimization research on AI policy and regulatory texts for smart healthcare, thereby deepening technological innovation and theoretical research in AI in smart healthcare.

## 3 Research design

### 3.1 Data sources and processing

The Chinese healthcare AI policy system adopts the model of framework guidance integrated with local adaptation, rather than mere duplication. Higher-level policies such as national policies focus on top-level design. They establish the overall principles, strategic direction and core goals to form the basic framework of the policy system. In contrast, policies at lower levels such as provincial and municipal levels, take into account regional resource endowments, medical development levels, and actual needs to supplement the specific implementation details and operational arrangements. The overlapping of administrative levels is limited to the core strategic consensus, but there are significant differences in the implementation paths, division of responsibility entities, and direction of resource allocation at the execution level. Therefore, adopting the multi-level policy approach is of utmost importance. The multi-level coverage ensures the comprehensiveness of the policy system, thereby providing the solid empirical foundation for subsequent analysis of policy quality differences and the formulation of targeted optimization suggestions.

Policy texts primarily originate from the official websites of the Central People’s Government of the People’s Republic of China, provincial-level people’s governments, the National Health Commission, the National Development and Reform Commission, and other relevant departments, as well as the Peking University Law Database. The research set the subject term “smart healthcare” and “artificial intelligence in healthcare” to search for policy texts. Adhering to the principles of openness, validity, relevance, completeness, and uniqueness in policy selection (30). This study used the following principles in the filtration of the documents: (1) selection of healthcare AI policies at the national, provincial, and city level; (2) policy text directly related to AI in smart healthcare; (3) policy issued from January 2015 to October 2025; (4) removal of policy documents that are no longer effective and have been revised. We selected the most representative 77 policy texts closely related to AI in smart healthcare through information searches and manual reading, including 20 at the national level, 19 at the provincial level, 7 at the municipal level, and 31 at the prefecture-level city level, demonstrating broad coverage across administrative tiers and extensive scope.

### 3.2 Construction of the PMC-Index model

**Figure 1:**
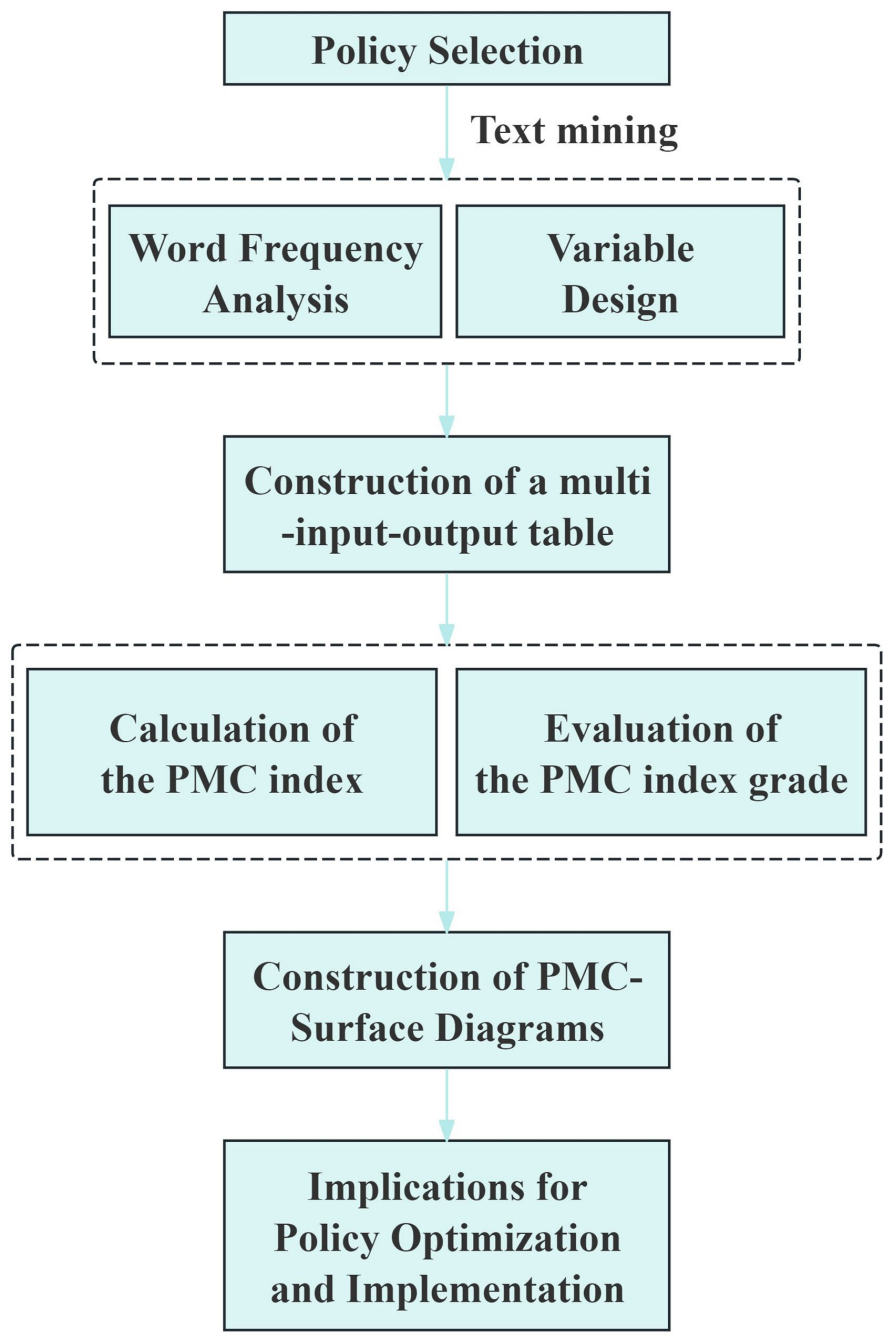
The specific analytical framework.

The PMC-Index model, a text-mining-based evaluation framework, primarily measures policy consistency and effectiveness. It originates from Ruiz Estrada’s “Everything in Motion” hypothesis, which posits that all elements in the universe are in constant motion and interconnected, thus assigning equal importance to any single variable. Compared to other quantitative policy evaluation methods, the PMC-Index model’s key advantage lies in its multidimensional assessment. By establishing two hierarchical levels of variables, it captures the diverse characteristics within policies, enabling more accurate evaluation of their strengths and weaknesses (31). The parameters of the PMC-Index model are primarily extracted through text mining, significantly reducing human subjectivity and enhancing the scientific rigor and rationality of quantitative evaluation. Furthermore, within the PMC-Index model, all variables carry equal weight, and no specific numerical values are assigned to variables during the evaluation process, effectively minimizing subjectivity in the assessment. The PMC-Index model is constructed in four steps: (1) excavate text and design the variables; (2) construct a multi-input–output table; (3) compute the index of PMC variables; (4) construct a PMC-Surface. The specific analytical framework is shown in Figure 1.

### 3.3 Word frequency analysis and variable design

#### 3.3.1 Word frequency analysis

**Figure 2:**
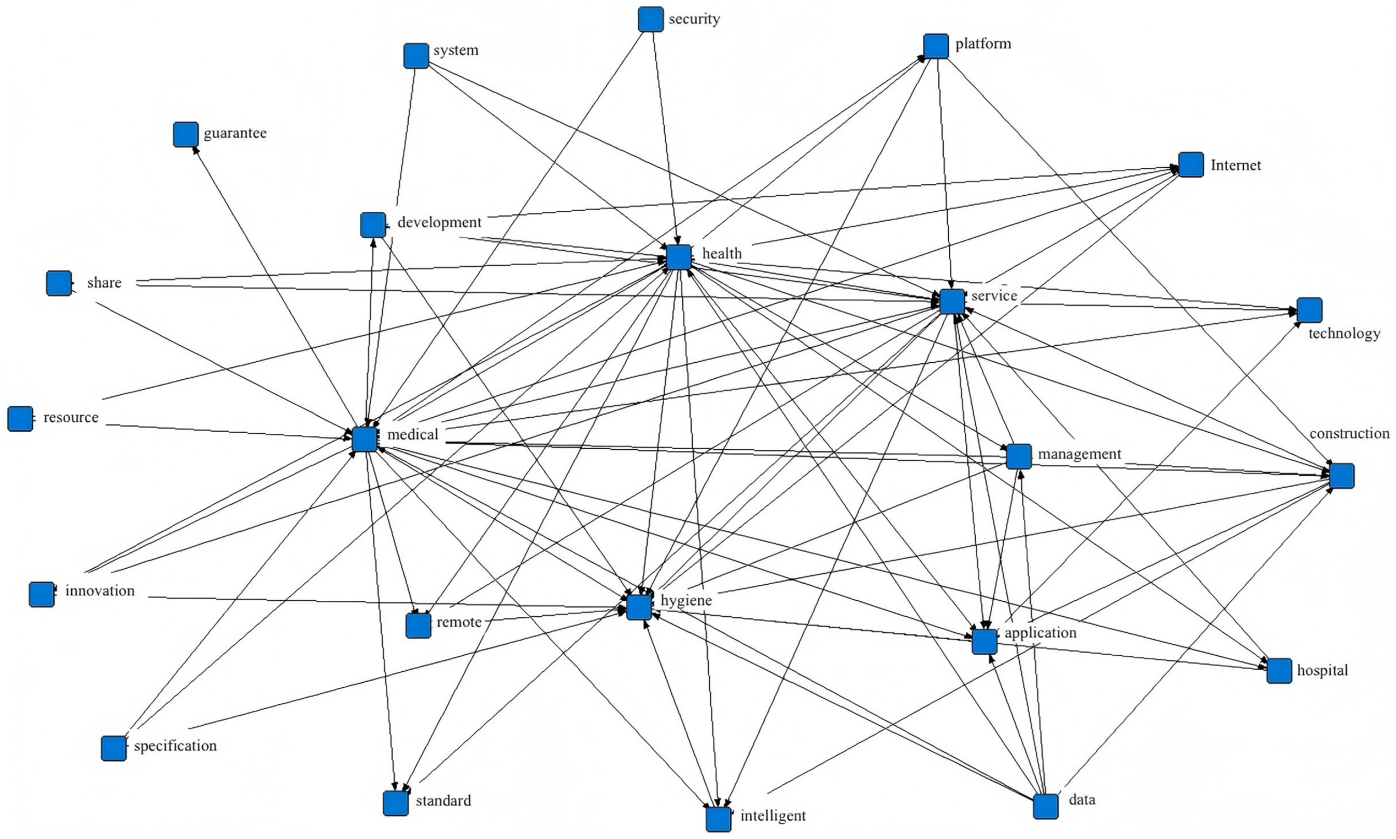
Semantic network diagram of high-frequency co-occurring terms in smart healthcare policy texts.

Analyzing and mining the policy text helps to accurately and scientifically refine the policy focus, thus providing a reference basis for indicator selection and variable design. This study employed the ROSTCM6 text mining software for information extraction from policy documents. First, 77 smart healthcare policy documents were consolidated into a single file and imported into ROSTCM6 for word segmentation and frequency analysis. Interfering terms such as “promote” and “strengthen” were filtered out, and synonyms were merged. High-frequency co-occurring terms were ultimately extracted, forming the semantic network diagram shown in Figure 2. The top 30 high-frequency terms were selected and compiled into a high-frequency term list (Table 1). Results indicate that core terms in smart healthcare policy texts include “medical care,” “health,” “services,” “data,” and “intelligence.” Related research directions span internet and informatization fields, while policy measures involve stakeholders such as hospitals, governments, and enterprises.

**Table 1:** High-frequency words statistics in policy texts.

Sequence	High frequency words	Frequency
1	Medical	6,075
2	Health	5,291
3	Service	3,846
4	Data	2,937
5	Intelligent	2,672
6	Hygiene	2,426
7	Management	1984
8	Internet	1977
9	Construction	1731
10	Application	1,688
11	Hospital	1,498
12	Development	1,398
13	Platform	1,326
14	Technology	1,114
15	System	1,043
16	Safety	958
17	Shared	784
18	Standard	762
19	Resources	729
20	Innovation	728
21	Guarantee	720
22	IT	673
23	standard	570
24	Remote	563
25	mechanism	516
26	Foundation	507
27	Government	477
28	Enterprise	477
29	Public	445
30	Clinical	440

In the semantic network diagram, high-frequency terms within policy texts are interconnected through network links, visually illustrating the relationships between these frequently occurring words. High-frequency terms are represented as nodes: nodes with numerous connections to other nodes exhibit higher frequency and broader reach, indicating greater importance. Keywords such as “medical care,” “health,” “hygiene,” “services,” and ‘intelligent’ demonstrate high centrality. Among these, the keyword “health,” central to the medical and healthcare sector, occupies a pivotal position in the diagram with extensive influence. This indicates that healthcare-related work extends beyond the ultimate goal of “health,” encompassing diverse operational aspects of the healthcare system such as “medical” service provision, “sanitation” safeguards, and ‘smart’ optimization. The interconnections among high-frequency terms like “platform,” “internet,” “technology,” “data,” “construction,” “development,” and “application” also reflect the series of supporting measures provided for healthcare advancement through technological means and platform development.

#### 3.3.2 Variable design

Based on the statistical analysis of high-frequency words in policy texts and semantic network analysis, combined with text mining of smart healthcare policies, this paper ultimately constructs a smart healthcare policy evaluation index system that includes 9 primary variables such as policy nature, policy field, policy timeliness, policy objectives, and policy content, and 35 secondary variables such as industrial innovation, model promotion, standard establishment, platform construction, and resource sharing. Each secondary indicator is assigned equal weight, and parameter values are set using a binary system: if a draft text contains content corresponding to the meaning of a secondary variable, the parameter is marked as 1; otherwise it is marked as 0, as shown in Table 2.

**Table 2:** Smart healthcare policy evaluation indicator system.

Primary variables	Secondary variables	Meaning of secondary variable
Policy nature (X_1_)	X_11_Regulation	Whether the policy contains regulatory content: Yes = 1, No = 0
	X_12_Prediction	Whether the policy contains predictive content: Yes = 1, No = 0
	X_13_Guidance	Whether the policy contains guiding content: Yes = 1, No = 0
Policy domain (X_2_)	X_21_Economy	Whether the policy involves the economic sector: Yes = 1, No = 0
	X_22_Health	Whether the policy involves the health sector: Yes = 1, No = 0
	X_23_Technology	Whether the policy involves the science and technology sector: Yes = 1, No = 0
Policy timeliness (X_3_)	X_31_Long-term	Whether the policy mentions the content of more than 5 years: Yes = 1, No = 0
	X_32_Medium-term	Whether the policy mentions the content of 3–5 years: Yes = 1, No = 0
	X_33_Short-term	Whether the policy mentions the content within 3 years: Yes = 1, No = 0
Policy objectives (X_4_)	X_41_Industrial Innovation	Whether policy objectives include industrial innovation: Yes = 1, No = 0
	X_42_Model Promotion	Whether policy objectives include model promotion: Yes = 1, No = 0
	X_43_Standard Establishment	Whether policy objectives include standard establishment: Yes = 1, No = 0
	X_44_Platform Development	Whether policy objectives include platform construction: Yes = 1, No = 0
	X_45_Resource Sharing	Whether policy objectives include resource sharing: Yes = 1, No = 0
Policy content (X_5_)	X_51_Infrastructure Development	Whether the content of the policy involves infrastructure construction: Yes = 1, No = 0
	X_52_Service Model Innovation	Whether the content of the policy involves service model innovation: Yes = 1, No = 0
	X_53_Safety Management Assurance	Whether the content of the policy involves safety management assurance: Yes = 1, No = 0
	X_54_Application Scenario Promotion	Whether the content of the policy involves application scenario promotion: Yes = 1, No = 0
Policy object (X_6_)	X_61_Government	Whether the policy object includes the government: Yes = 1, No = 0
	X_62_Enterprise	Whether the policy object includes enterprise: Yes = 1, No = 0
	X_63_Community	Whether the policy object includes the community: Yes = 1, No = 0
	X_64_Family	Whether the policy object includes family: Yes = 1, No = 0
Policy evaluation (X_7_)	X_71_Sufficient basis	Whether the policy basis is sufficient: Yes = 1, No = 0
	X_72_Clear objectives	Whether the policy objectives are clear: Yes = 1, No = 0
	X_73_Detailed program	Whether the policy program is detailed: Yes = 1, No = 0
	X_74_Scientific planning	Whether the policy planning is scientific: Yes = 1, No = 0
Policy measures (X_8_)	X_81_Funding Support	Whether the policy includes funding support: Yes = 1, No = 0
	X_82_Talent Development	Whether the policy includes talent development: Yes = 1, No = 0
	X_83_Legal Safeguards	Whether the policy includes legal safeguards: Yes = 1, No = 0
	X_84_Assessment and Evaluation	Whether the policy includes assessment and evaluation: Yes = 1, No = 0
	X_85_Demonstration and Promotion	Whether the policy includes demonstration and promotion: Yes = 1, No = 0
Policy tool (X_9_)	X_91_Mandatory	Whether the policy is mandatory: Yes = 1, No = 0
	X_92_Innovative	Whether the policy is innovative: Yes = 1, No = 0
	X_93_Market-oriented	Whether the policy is market-oriented: Yes = 1, No = 0
	X_94_Collaborative	Whether the policy is collaborative: Yes = 1, No = 0

### 3.4 Constructing a multi-input–output table

Based on the construction principles of the PMC-Index model and in conjunction with the variable settings of the Smart Healthcare PMC-Index Model, a multi-input–output table was established, as shown in Table 3.

**Table 3:** Multi-input–output table.

Primary variables	Secondary variables
X_1_	X_11_X_12_X_13_
X_2_	X_21_X_22_X_23_
X_3_	X_31_X_32_X_33_
X_4_	X_41_X_42_X_43_X_44_X_45_
X_5_	X_51_X_52_X_53_X_54_
X_6_	X_61_X_62_X_63_X_64_
X_7_	X_71_X_72_X_73_X_74_
X_8_	X_81_X_82_X_83_X_84_X_85_
X_9_	X_91_X_92_X_93_X_94_

### 3.5 PMC-index calculation and evaluation grade classification

The calculation of the PMC-Index can be divided into three steps: (1) Set the secondary variable values based on Equations 1, 2, and construct a multi-input–output table; (2) Calculate the primary variable values based on Equation 3; (3) Calculate the PMC index based on Equation 4.


(1) X~N[0,1]

(2) X={XR:[0,1]}

(3) $$X_{i}(\sum_{j=1}^{n}\frac{X_{\mathrm{ij}}}{T(X_{\mathrm{ij}})}),i=1,2,3,n\;\;$$

(4) $$\mathrm{PMC}=[\begin{array}{c} X_{1}(\sum_{j=1}^{3}\frac{X_{1j}}{3})+X_{2}(\sum_{j=1}^{3}\frac{X_{2j}}{3})+X_{3}(\sum_{j=1}^{3}\frac{X_{3j}}{3})+\\ X_{4}(\sum_{j=1}^{5}\frac{X_{4j}}{5})+X_{5}(\sum_{j=1}^{4}\frac{X_{5j}}{4})+X_{6}(\sum_{j=1}^{4}\frac{X_{6j}}{4})+\\ X_{7}(\sum_{j=1}^{4}\frac{X_{7j}}{4})+X_{8}(\sum_{j=1}^{5}\frac{X_{8j}}{5})+X_{9}(\sum_{j=1}^{4}\frac{X_{9j}}{4}) \end{array}]$$

Among these,i is a first-order variable;jis a second-order variable;n is the number of secondary variables corresponding to the primary variable i, j,n = 1, 2, 3, 4, ….

### 3.6 Construction of PMC-surface diagrams

PMC-Surface plots visually represent model calculation results, intuitively illustrating the strengths and weaknesses of policy samples. Their construction primarily relies on the PMC matrix, composed of nine primary policy variables as defined in Equation 5.


(5) $$\mathrm{PMC}-\text{Surface}=[\begin{array}{c} X_{1}\;\;\;X_{2\;\;\;}\;X_{3}\\ X_{4}\;\;\;X_{5\;\;\;}\;X_{6}\\ X_{7}\;\;\;X_{8\;\;\;}\;X_{9} \end{array}]$$

## 4 Quantitative analysis of smart healthcare policies

### 4.1 Selection of representative policies

Through textual analysis of 77 smart healthcare policies, we selected 10 representative policies for analysis and evaluation. These policies are cutting-edge, all issued between 2024 and 2025. They cover a broad and comprehensive range of levels, involving the national government, various provinces, and multiple cities. The content includes medical support and security systems, data element evaluation, technology development and application, and other areas closely related to research in the smart healthcare field, as detailed in Table 4.

**Table 4:** Representative policies for smart healthcare.

Item	Policy name	Date of release
P1	Notice on the Three-Year Action Plan for Enhancing Smart Hospital Capabilities in Tianjin Municipality (2024–2026)	28 May 2024
P2	Several Opinions on Promoting the Development of Digital Traditional Chinese Medicine	19 July 2024
P3	Notice on the Reference Guidelines for Artificial Intelligence Application Scenarios in the Health Sector	6 November 2024
P4	Shanghai Work Plan for Developing Medical Artificial Intelligence (2025–2027)	23 November 2024
P5	Specifications for Information and Digitalization Construction in Traditional Chinese Medicine Hospitals (2024 Edition)	5 December 2024
P6	Chongqing Action Plan for Innovative Development of Smart Medical Equipment Industry (2025–2027)	6 January 2025
P7	Implementation Plan for Digital and Intelligent Transformation of the Pharmaceutical Industry (2025–2030)	3 April 2025
P8	Beijing Action Plan for Accelerating Innovation in “AI + Pharmaceutical Health” (2025–2027)	3 July 2025
P9	Notice on Zhejiang Province’s Action Plan for Accelerating High-Quality Development of “AI + Healthcare” (2025–2027)	11 July 2025
P10	Hubei Implementation Plan for Accelerating AI Application in Healthcare (2025–2027)	18 July 2025

### 4.2 PMC-index of 10 representative policies

Following the calculation steps of the aforementioned PMC-Index model, content analysis and text mining methods were employed to assign values to the secondary variable parameters in the input–output tables of the 10 smart healthcare policies. This ultimately established the input–output tables for the 10 smart healthcare policies, as shown in Table 5.

**Table 5:** Multi-input–output table of 10 representative policies.

Primary variables	Secondary variables	P1	P2	P3	P4	P5	P6	P7	P8	P9	P10
X_1_	X_11_	1	1	1	1	1	1	1	1	1	1
	X_12_	1	1	0	0	0	1	1	1	1	1
	X_13_	1	1	1	1	1	1	1	1	1	1
X_2_	X_21_	1	1	1	1	1	1	1	1	1	1
	X_22_	1	1	1	1	1	1	1	1	1	1
	X_23_	1	1	1	1	1	1	1	1	1	1
X_3_	X_31_	0	0	1	0	1	0	1	0	0	0
	X_32_	1	1	1	1	1	1	1	1	1	1
	X_33_	0	0	0	0	0	0	0	0	0	0
X_4_	X_41_	0	1	1	1	1	1	1	1	1	0
	X_42_	1	1	1	0	1	0	1	0	0	0
	X_43_	1	1	1	1	1	1	1	1	1	0
	X_44_	1	1	1	1	1	1	1	1	1	1
	X_45_	1	1	1	1	1	1	1	1	1	1
X_5_	X_51_	1	1	1	1	1	0	1	1	1	1
	X_52_	1	1	1	1	1	1	1	1	1	1
	X_53_	1	1	1	0	1	0	1	0	1	1
	X_54_	0	1	1	1	0	1	1	1	1	1
X_6_	X_61_	1	1	1	1	1	1	1	1	1	1
	X_62_	1	1	1	1	1	1	1	1	1	1
	X_63_	1	1	1	1	1	1	1	1	1	1
	X_64_	1	1	1	1	1	1	1	1	1	1
X_7_	X_71_	1	1	1	1	1	1	1	1	1	1
	X_72_	1	1	1	1	1	1	1	1	1	1
	X_73_	1	1	1	1	1	1	1	1	1	1
	X_74_	1	1	1	1	1	1	1	1	1	1
X_8_	X_81_	1	0	0	1	0	1	0	0	1	0
	X_82_	1	1	1	1	0	1	1	1	1	0
	X_83_	0	0	0	0	1	1	1	1	0	0
	X_84_	1	1	1	1	1	0	1	1	1	1
	X_85_	0	1	0	0	1	1	1	0	0	1
X_9_	X_91_	1	1	1	1	1	1	1	1	1	1
	X_92_	1	1	1	1	1	1	1	1	1	1
	X_93_	0	0	1	0	1	0	0	0	0	1
	X_94_	0	1	1	1	0	1	1	1	1	1

Based on a multi-input–output table of 10 smart healthcare policies, the PMC-Index for each policy was calculated, as shown in Table 6.

**Table 6:** PMC-Index of 10 representative policies.

Item	P1	P2	P3	P4	P5	P6	P7	P8	P9	P10	Average
X1	1.00	1.00	0.67	0.67	0.67	1.00	1.00	1.00	1.00	1.00	0.90
X2	1.00	1.00	1.00	1.00	1.00	1.00	1.00	1.00	1.00	1.00	1.00
X3	0.33	0.33	0.67	0.33	0.67	0.33	0.67	0.33	0.33	0.33	0.43
X4	0.80	1.00	1.00	0.80	1.00	0.80	1.00	0.80	0.80	0.40	0.84
X5	0.75	1.00	1.00	0.75	0.75	0.50	1.00	0.75	1.00	1.00	0.85
X6	1.00	1.00	1.00	1.00	1.00	1.00	1.00	1.00	1.00	1.00	1.00
X7	1.00	1.00	1.00	1.00	1.00	1.00	1.00	1.00	1.00	1.00	1.00
X8	0.60	0.60	0.40	0.60	0.60	0.80	0.80	0.60	0.60	0.40	0.60
X9	0.50	0.75	1.00	0.75	0.75	0.75	0.75	0.75	0.75	1.00	0.78
PMC-Index	6.98	7.68	7.73	6.90	7.43	7.18	8.22	7.23	7.48	7.13	7.40
Rank	9	3	2	10	5	7	1	6	4	8	

Policy nature (X1): The mean value of policy attribute X1 is 0.90, indicating that the selected smart healthcare policies involve regulatory and guiding elements, while few policies lack predictions for the future development outcomes of smart healthcare.

Policy domain (X2): X2 have the highest average scores of 1, indicating that all 10 smart healthcare policies involve the fields of economy, health, and technology, taking into account the multidimensional aspects of smart healthcare in social development.

Policy timeliness (X3): X3 has the lowest score of 0.43, indicating that many policies fail to combine long-term, medium-term, and short-term goals, suggesting that most smart healthcare policies tend to prioritize immediate goals at the expense of long-term goals.

Policy objectives (X4): X4 has a score of 0.84, indicating that the comprehensiveness of objectives is generally considered in policy formulation, but there are deficiencies in promotion.

Policy content (X5): X5 has a score of 0.85, indicating that policy content covers infrastructure, service models, and security management.

Policy object (X6): The mean value of policy attribute X6 is 1, demonstrating that the policies involve a wide range of targets, fully leveraging the roles of various policy entities in smart healthcare.

Policy evaluation (X7): The mean value is the same as X1 and X6, which is 1, indicating that the policies are well-founded and have clear planning, demonstrating a certain degree of scientificity and feasibility.

Policy measures (X8): X8 has a score of 0.6, ranking second to last, indicating that efforts in financial support and legal protection are relatively low, with only a small number of policies covering these aspects. This suggests that these two aspects have not been fully considered in the formulation of smart healthcare policies and need to be strengthened.

Policy tools (X9): X9 has a score of 0.78, indicating that the selected smart healthcare policies focus on mandatory, innovative, and collaborative tools, but only three policies involve market tools, showing a lack of application.

Subsequently, based on the PMC index scores of each policy and referring to previous literature (32, 33), this study categorizes the policies into four grades: poor [0–2), fair [2–4), good [4–6), outstanding [6, 8) and excellent [8, 9), as shown in Table 7.

**Table 7:** Policy sample rating based on the PMC index model.

Score range	[8,9]	[6,8)	[4,6)	[2,4)	[0,2)
Level	(A) Excellent	(B) Outstanding	(C) Good	(D) Fair	(E) Poor

The average PMC-Index for the smart healthcare policy samples is 7.40, placing them in the excellent category of policy ratings. This indicates overall high quality with a degree of scientific rigor and rationality. Specifically, the ranking of the 10 smart healthcare policies is as follows: P7 > P3 > P2 > P9 > P5 > P8 > P6 > P10 > P1 > P4. Among these, one policy sample achieved a perfect rating, while nine policy samples attained an excellent rating.

**Figure 3:**
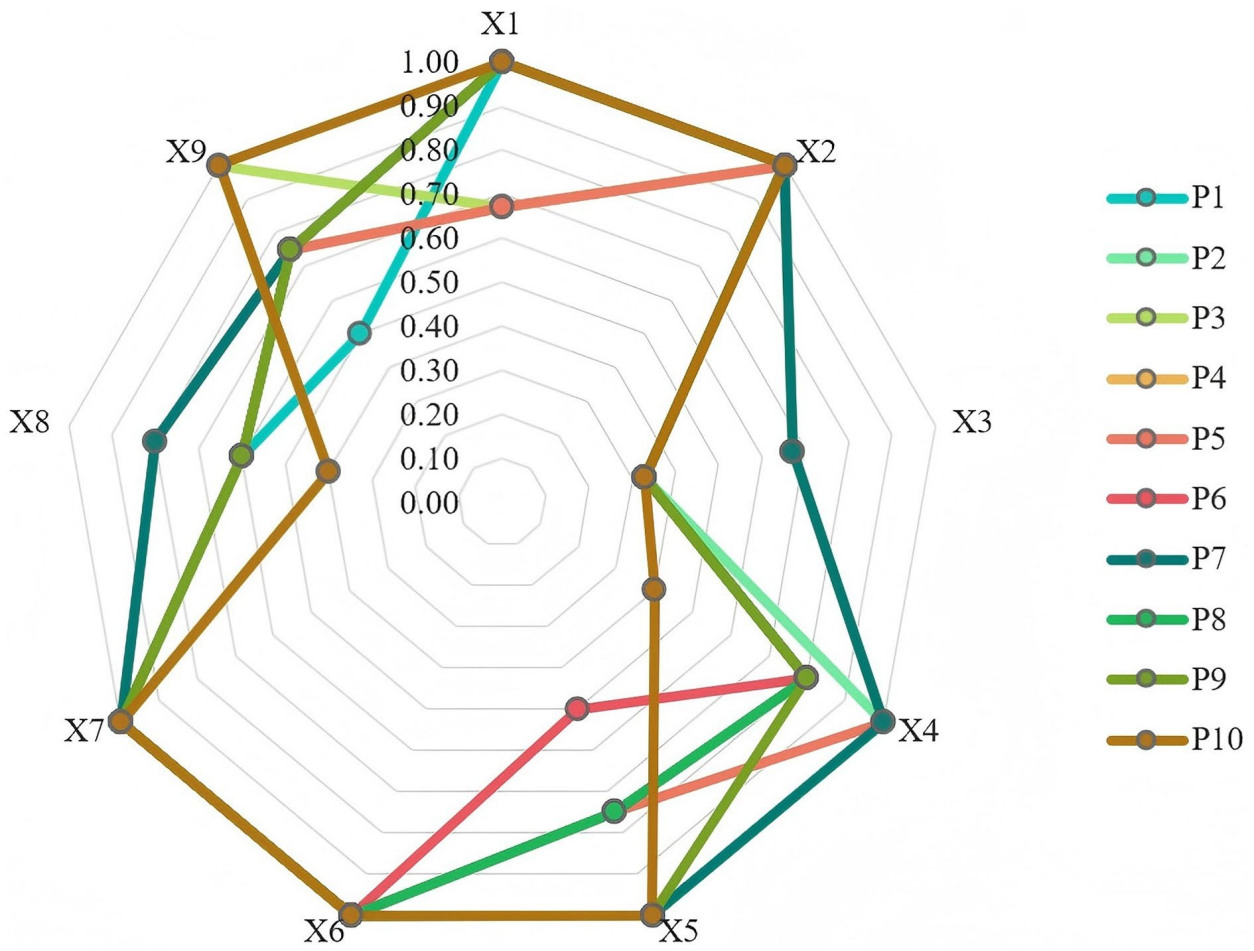
Debra chart of 10 representative policies.

To provide a more intuitive visualization of the overall scores for each smart healthcare policy, a radar chart was created. By comparing the average values of key variables across policies, it presents the overall score trends for 10 representative policies, as shown in Figure 3.

The primary variables exhibiting significant variation are policy objectives (X4), policy content (X5), policy measures (X8), and policy instruments (X9). Based on the score distribution, these four variables demonstrate marked differences across the ten policies. Some policies achieved near-perfect scores in these areas, while others scored well below the average. Taking policy objectives (X4) as an example, some focus on specific areas, such as smart healthcare sub-scenarios and regional medical collaboration policies. They score higher due to precise targeting and strong alignment with actual needs, whereas some policies with a broader scope score slightly lower because their objectives are relatively general. Variations in policy content (X5) were evident in the level of detail and specificity. Policies addressing local or industry pain points by refining technical applications and resource allocation received higher scores, while others leaning toward framework guidance with insufficient detail scored lower. Differences in policy measures (X8) primarily stemmed from their operational feasibility. Policies clearly defining responsible entities, implementation steps, and timelines demonstrated strong practicality, while others scored lower due to overly general measures lacking concrete implementation pathways. Changes in policy instruments (X9) are reflected in the diversity and adaptability of tool selection. Some policies comprehensively employ multiple instruments such as fiscal support, technical standards, and supervision and assessment, achieving high alignment with policy objectives. Conversely, certain policies overly rely on a single instrument, demonstrating insufficient flexibility in tool combinations and consequently receiving relatively lower scores. This significant variation fundamentally reflects the different policy adjustments made to address differentiated needs, as well as the distinct approaches taken by policymakers in setting objectives, planning content, designing measures, and selecting tools.

The policy nature (X1) and policy timeliness (X3) variables exhibit relatively minor variations. Most policies score near the average on these two metrics, showing no significant divergence in scores. Regarding policy nature (X1), this indicates that all policies maintain a high degree of consistency in their typological definitions. There are no substantial adjustments to their nature due to differences in policy objectives or application scenarios. This allows policy recipients to clearly grasp the core positioning and implementation orientation of the policies, avoiding execution deviations caused by ambiguous nature. The minimal variation in policy timeliness (X3) indicates relative uniformity in planning temporal dimensions such as validity periods and phase divisions. This avoids both the issue of overly short timelines causing rushed implementation and the problem of excessively long durations lacking flexibility. Such stability fosters a continuous and coherent policy implementation environment, enabling stakeholders to formulate long-term execution plans based on clear timelines. This reduces resource wastage and planning confusion caused by frequent adjustments to policy timelines. However, this minimal variation warrants scrutiny regarding alignment with dynamic practical demands. Subsequent adjustments should be made to the policy’s nature and the reasonableness of its timing in light of the industry’s development pace and external environmental changes, so as to better meet the actual development needs.

The primary variables that remained largely unchanged were policy domains (X2) and policy evaluation (X7). All ten policies achieved full marks on both variables X2 and X7, indicating that these policies consistently focused on core domains without deviating from their primary objectives. This also demonstrates that the policy evaluation system was well-designed, featuring clear evaluation criteria and standardized evaluation procedures.

### 4.3 Analysis of PMC-surface for 10 representative policies

**Figure 4:**
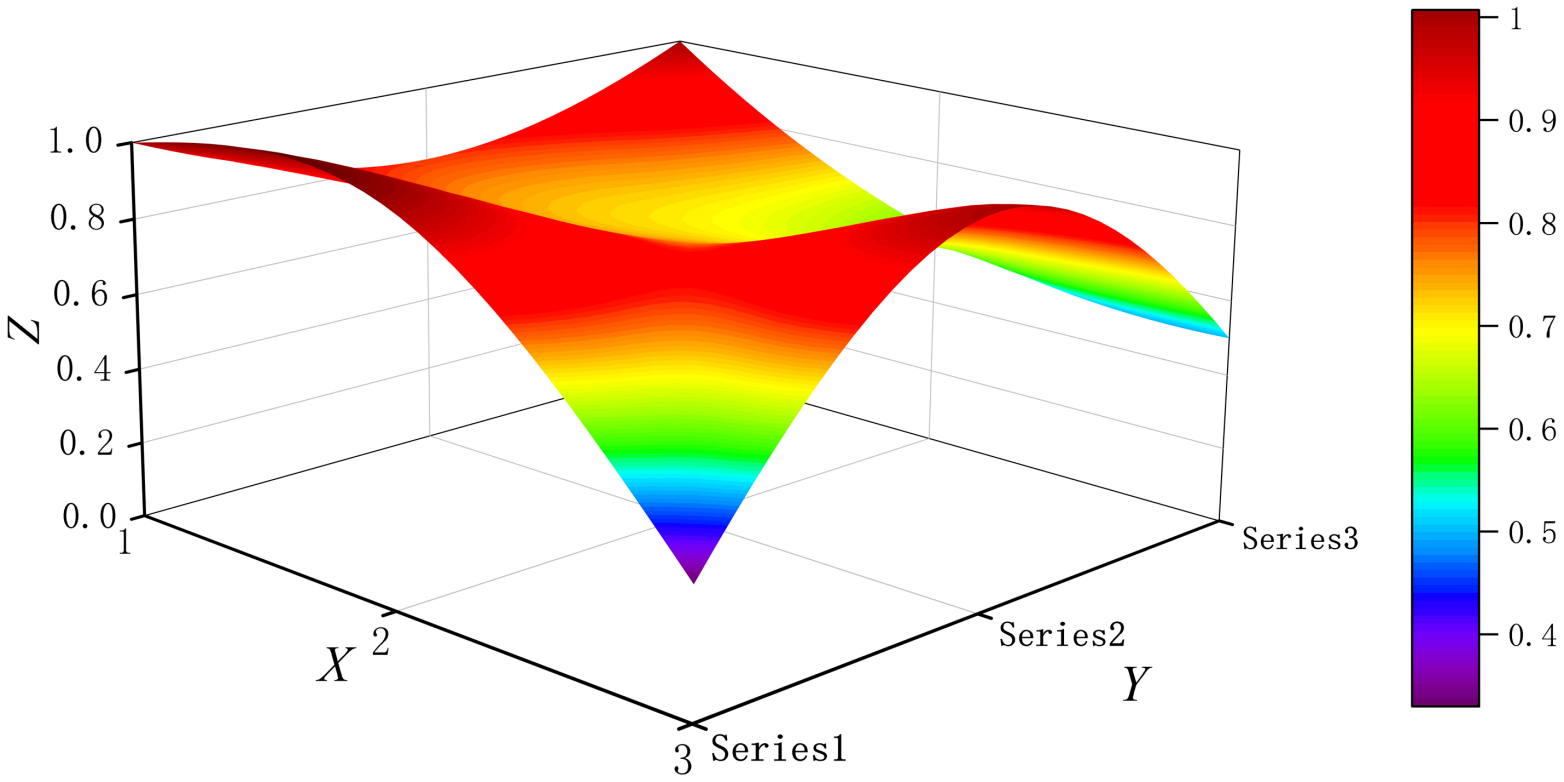
The PMC-Surface for P1.

**Figure 5:**
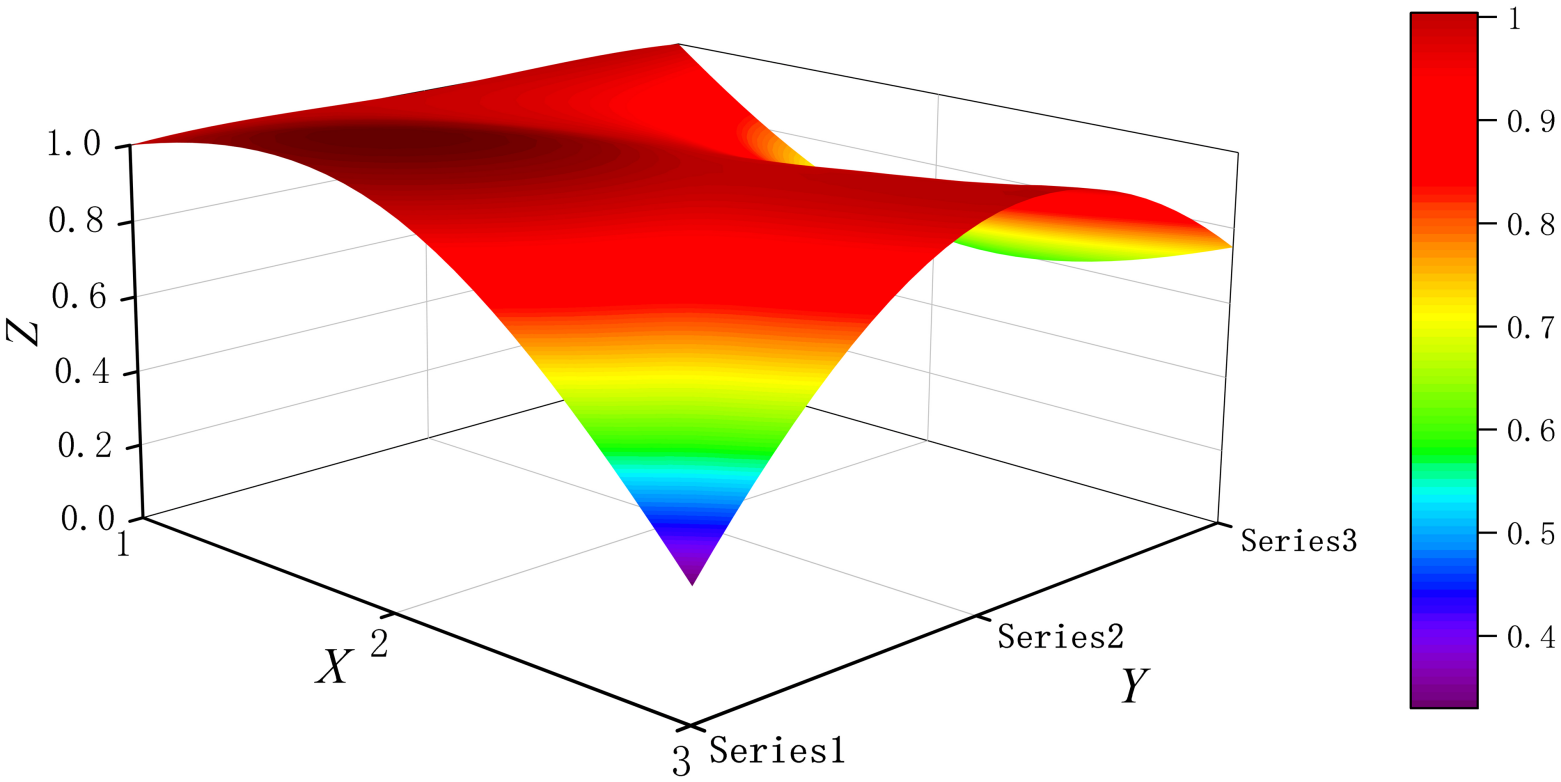
The PMC-Surface for P2.

**Figure 6:**
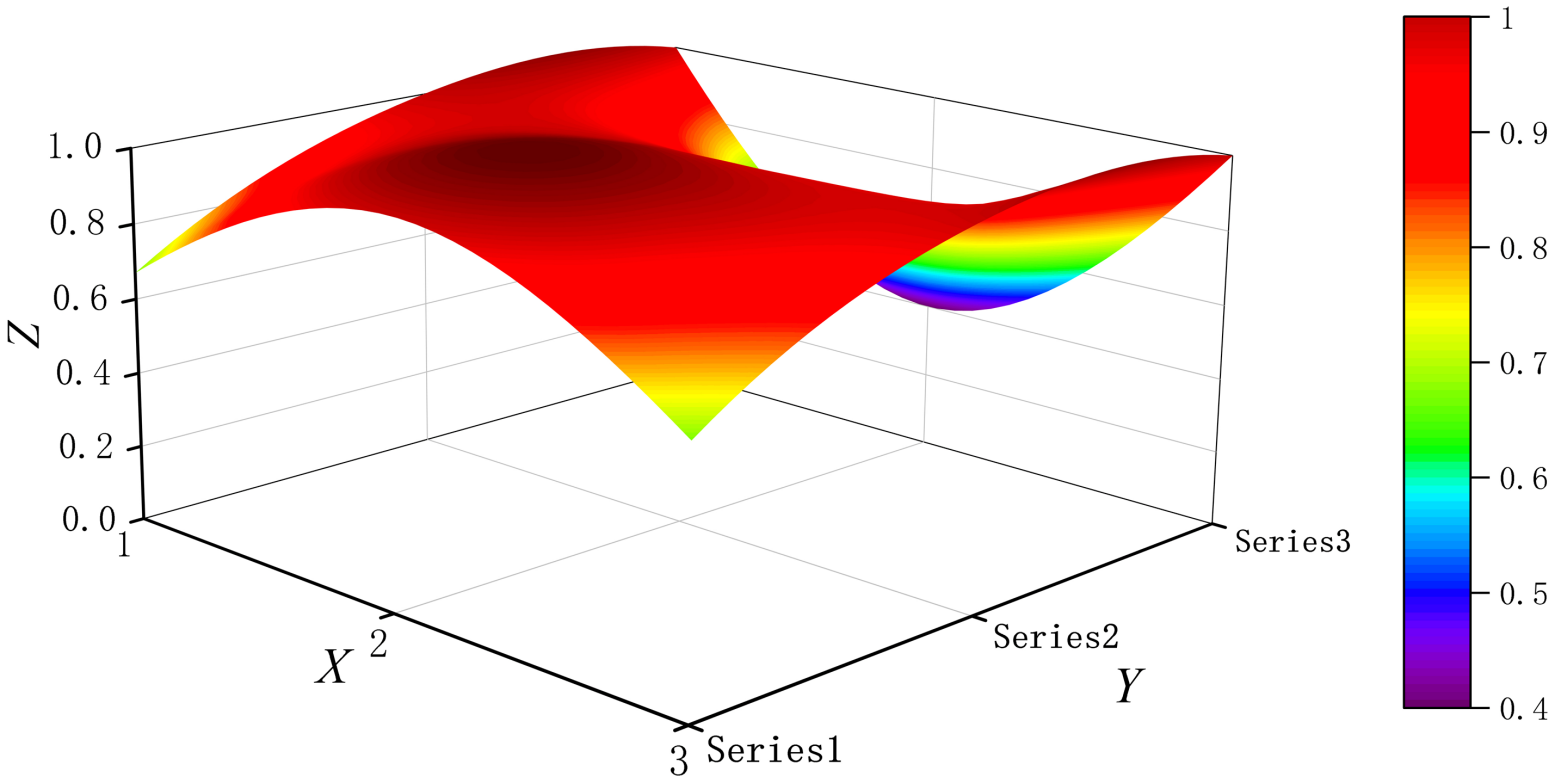
The PMC-Surface for P3.

**Figure 7:**
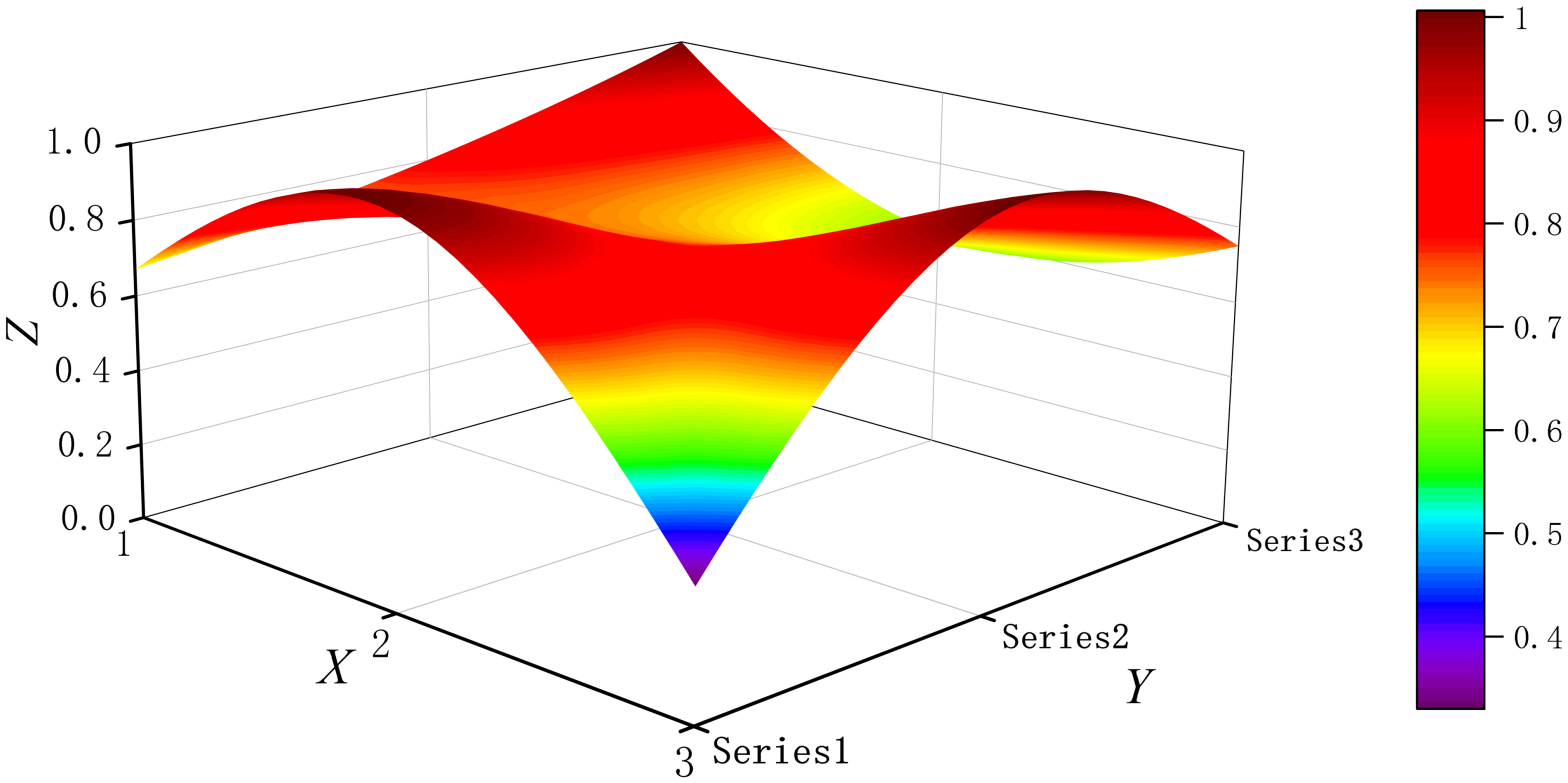
The PMC-Surface for P4.

**Figure 8:**
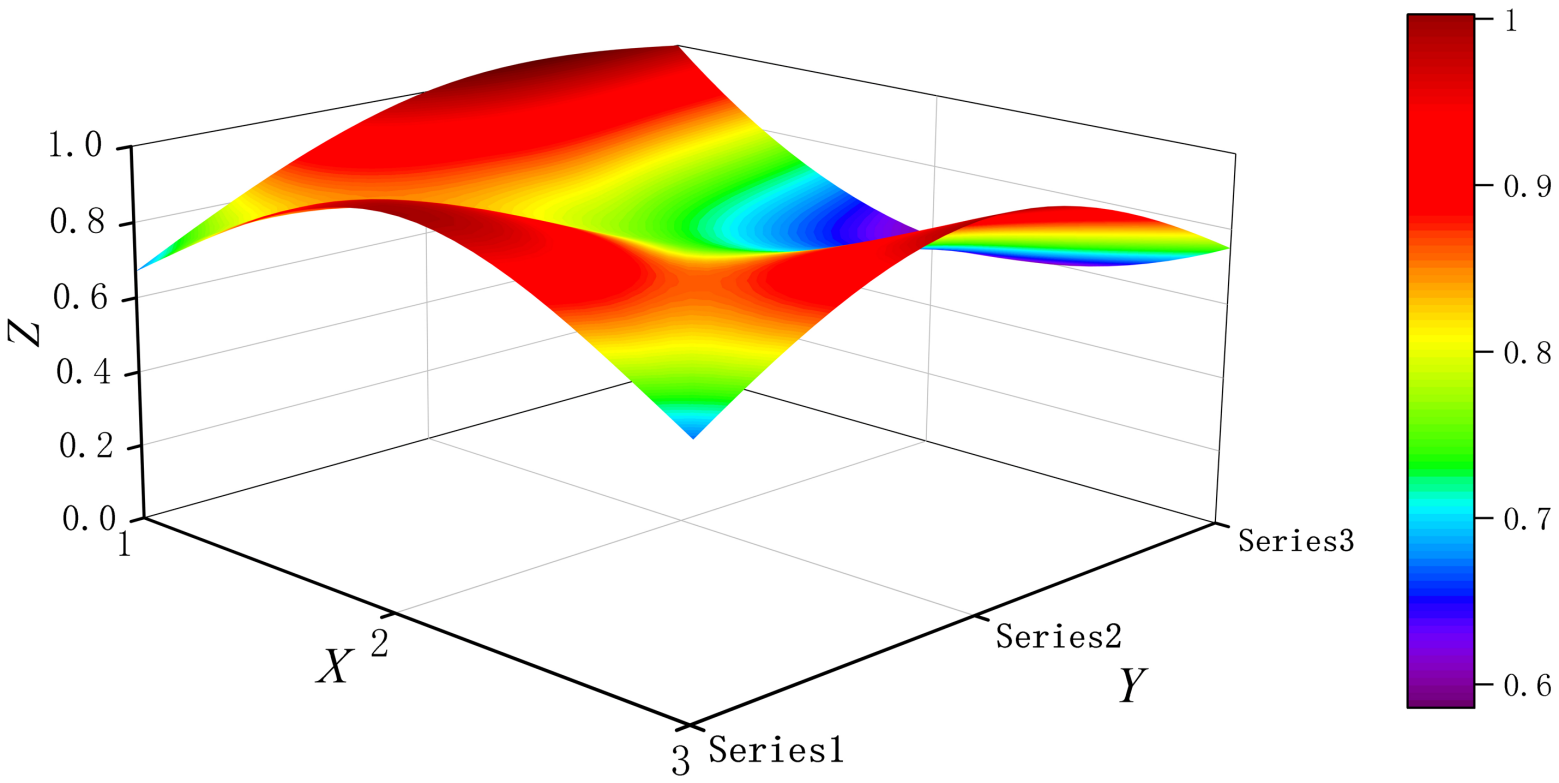
The PMC-Surface for P5.

**Figure 9:**
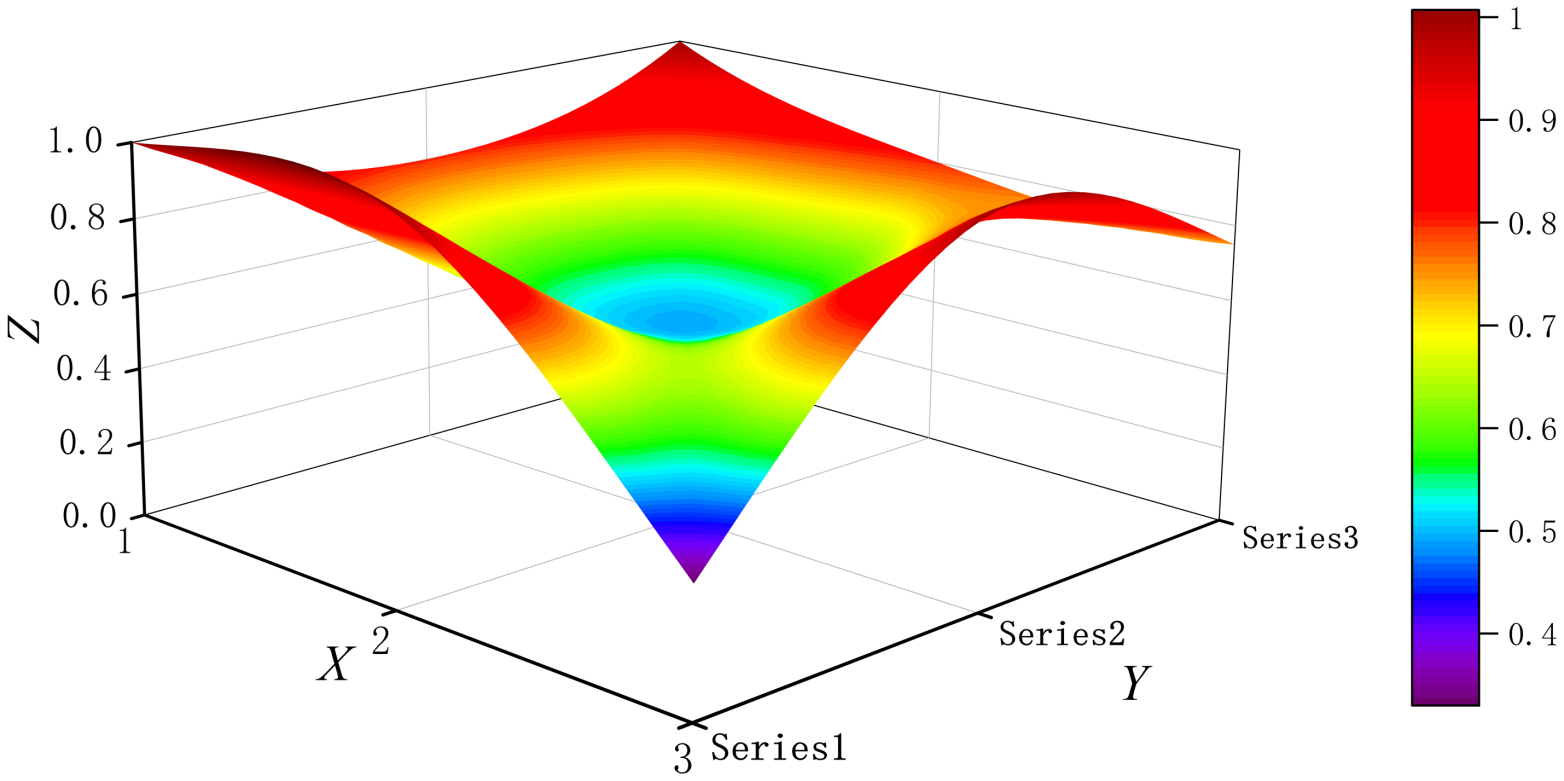
The PMC-Surface for P6.

**Figure 10:**
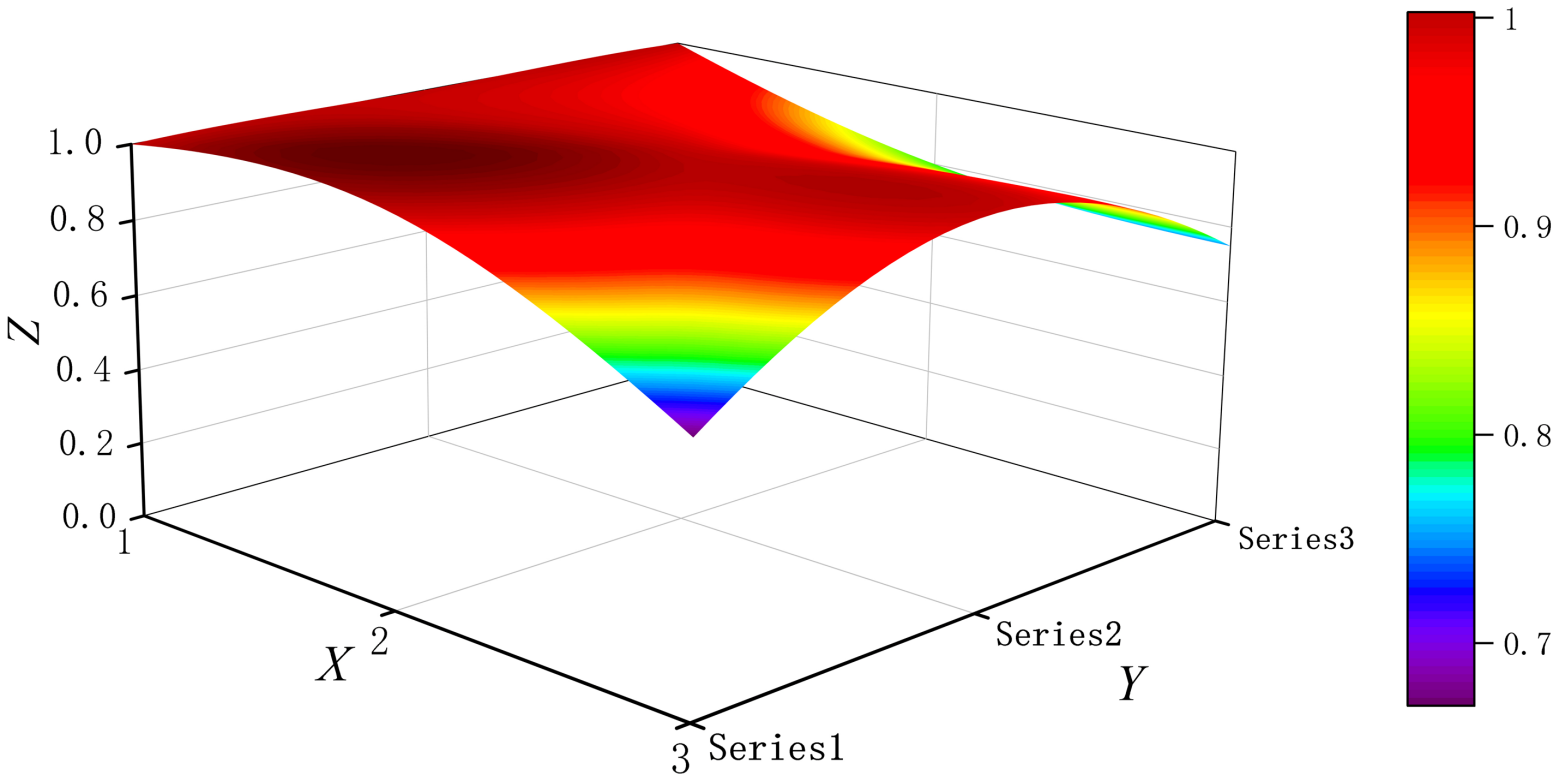
The PMC-Surface for P7.

**Figure 11:**
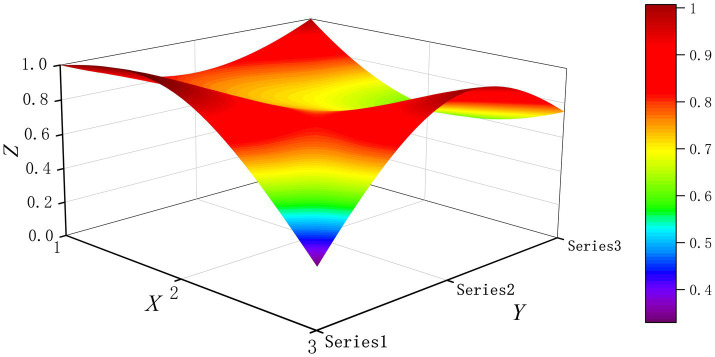
The PMC-Surface for P8.

**Figure 12:**
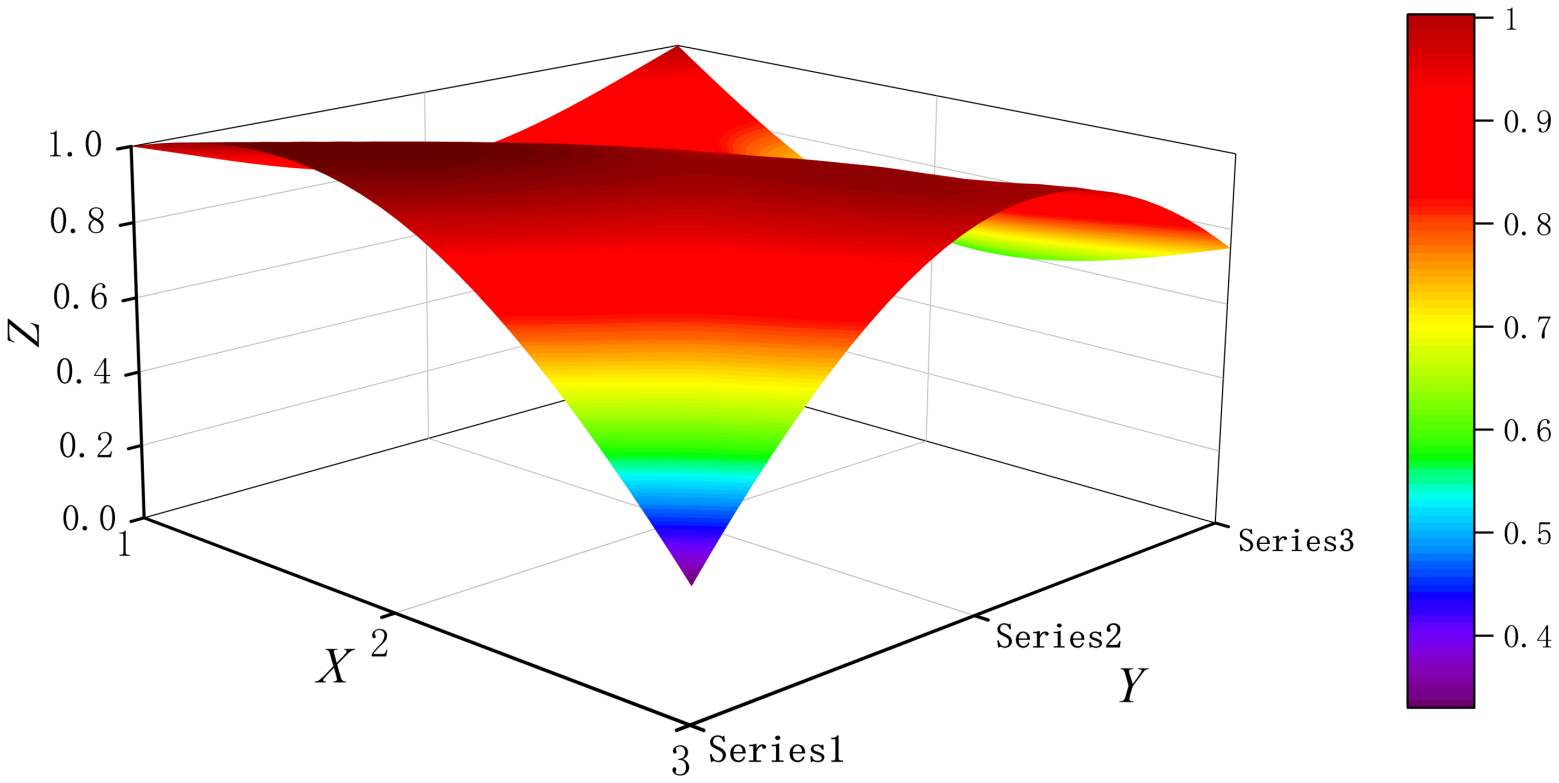
The PMC-Surface for P9.

**Figure 13:**
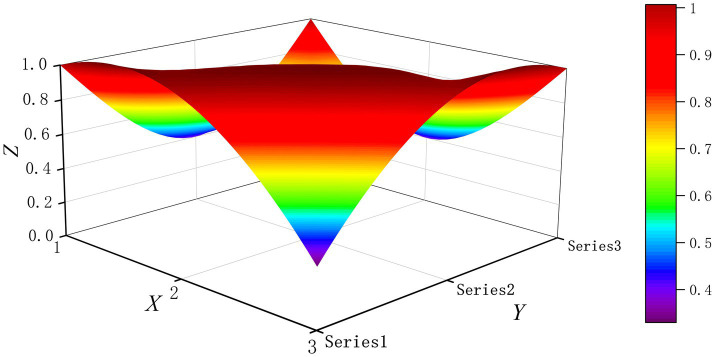
The PMC-Surface for P10.

The PMC-Surface plot is constructed using the PMC matrix Equation 5, forming an irregular three-dimensional uneven surface where different color blocks represent varying indicator scores. To compare the PMC-Index results across policy and regulatory texts, this paper utilizes MATLAB software to plot PMC-Surface diagrams showing peak PMC indices for each category, as illustrated in Figures 4–13. (Series 1: X1 policy nature, X2 policy domain, X3 policy timeliness; Series 2: X4 policy objectives, X5 policy content, X6 policy object; Series 3: X7 policy evaluation, X8 policy measures, X9 policy tool.) Here, the X-axis represents matrix columns, the Y-axis represents matrix rows, and the Z-axis represents individual PMC indicator scores. The color and position within the PMC-Surface diagram indicate that larger red areas and values closer to 1 signify higher scores for the corresponding evaluation indicator, reflecting a higher overall consistency level of the policy or regulatory text. Conversely, values closer to 0 indicate lower scores for the corresponding evaluation indicator, with purple representing a lower consistency level of the policy or regulatory text.

By combining the PMC-Surface plots with the specific scores for each representative policy, we further analyzed the strengths and weaknesses of the policies and then proposed potential improvement paths for policy optimization. The specific analysis results are as follows.

Policy P1, “Notice on the Three-Year Action Plan for Enhancing Smart Hospital Capabilities in Tianjin (2024–2026),” scored 6.98 in the PMC-Index, earning an “Excellent” rating and ranking ninth. Issued by the Tianjin Municipal Health Commission, this policy aims to build upon prior achievements in smart healthcare development and further address “disconnects” and “bottlenecks” in hospital informatization upgrade. While indicators like X1 scored above average, all others were at or below the mean. This may stem from P1’s robust framework and foundational core attributes, yet showing weaknesses in practical implementation and innovation execution. As a specialized local government implementation plan, it features clear authority and legal legitimacy, combining administrative guidance with enforceable requirements, resulting in a solid baseline score. The low or average scores in other indicators may stem from: Policy objectives lack quantifiable metrics, reducing measurability; Policy content lacks scenario designs tailored to Tianjin’s context, resulting in insufficient depth and specificity; Policy measures fail to specify technical support details and fiscal subsidy ratios; Policy tools rely on traditional approaches, struggling to stimulate multi-stakeholder engagement, thereby lowering indicator scores and contributing to the overall low ranking. Recommended optimization path for indicators: X3-X9-X4-X8-X5.

Policy P2, “Several Opinions on Promoting the Development of Digital Traditional Chinese Medicine,” scored 7.68 on the PMC-Index, earning an “Excellent” rating and ranking third. The National Administration of Traditional Chinese Medicine introduced this policy to innovate development pathways that deeply integrate the inheritance of traditional Chinese medicine with digital technology. Only two indicators, X3 and X9, scored below average, indicating its distinct characteristics and strong synergy, but with deficiencies in timeliness and detail. Therefore, the recommended optimization path for indicators is X3-X9.

Policy P3, “Guidance on Artificial Intelligence Application Scenarios in the Health Sector,” scored 7.73 on the PMC-Index. This policy was rated “Excellent” and ranked second. Issued by the National Health Commission, it provides specific guidance for the rational and standardized application of AI technology in the health sector. Indicators X1 and X8 scored relatively low, likely due to excessive emphasis on guidance while lacking sufficient promotion and safeguards. Therefore, the recommended optimization path for indicators is X1-X8.

Policy P4, “Shanghai Medical Artificial Intelligence Development Plan (2025–2027),” scored 6.90 on the PMC-Index, earning an “Excellent” rating and ranking tenth. Issued by the Shanghai Municipal People’s Government, this policy aims to advance comprehensive development in Shanghai’s AI in smart healthcare sector while enhancing application capabilities and innovation capacity. Notably, the scores for all ten indicators were below or equal to the average level. This indicates that while the policy achieved an overall excellent rating, it lacks outstanding advantages in individual indicator performance. Its balanced approach fails to demonstrate distinct characteristics. The recommended optimization path is X3-X9-X4-X8-X5-X1.

Policy P5, “Specifications for Information and Digitalization Development in Traditional Chinese Medicine Hospitals (2024 Edition),” scored 7.43 on the PMC-Index, earning an “Excellent” rating and ranking fifth. The National Administration of Traditional Chinese Medicine has designated this policy as a key guideline for information and digitalization development in TCM hospitals. While indicators X1, X5, and X9 scored below average, the remaining six indicators met or exceeded standards. This discrepancy may stem from ambiguous cross-departmental coordination details, insufficient rigidity in policy implementation safeguards, content relevance and depth falling short of comparable exemplary policies, and limited diversity and innovation in policy tools. Accordingly, the recommended optimization path for indicators should be X1-X9-X5.

Policy P6, “Chongqing Smart Medical Equipment Industry Innovation and Development Action Plan (2025–2027),” scored 7.18 on the PMC-Index, earning an “Excellent” rating and ranking seventh. Issued by the Chongqing Municipal People’s Government, this policy aims to drive innovation and development in Chongqing’s smart medical equipment industry, enhance its core competitiveness, and accelerate its digital and intelligent transformation. Its scores for indicators X3, X4, X5, and X9 fell below the average level. It is recommended to adjust the optimization path for these indicators in the sequence X4-X3-X5-X9.

Policy P7, “Implementation Plan for Digital and Intelligent Transformation of the Pharmaceutical Industry (2025–2030),” scored 8.22 in the PMC-Index, earning a “Perfect” rating and ranking first. Issued by the Central Committee of the Communist Party of China and the State Council, this policy carries high authority. Except for indicator X9, all other indicators scored above or equal to the average, indicating the policy’s overall strong performance, significant advantages, and sound rationality. The optimization path for indicators is X9.

Policy P8, “Beijing Action Plan for Accelerating Innovation in ‘Artificial Intelligence + Healthcare’ (2025–2027),” scored 7.23 on the PMC-Index, earning an “Excellent” rating and ranking sixth. Five of its indicators achieved scores at or above the average level, indicating strong performance in these areas that positively supported the policy’s overall effectiveness. Indicators X3, X4, X5, and X9 scored below the policy’s average. Therefore, it is recommended to optimize and adjust indicators X4-X3-X5-X9.

Policy P9, “Notice on the Action Plan for Accelerating the High-Quality Development of ‘Artificial Intelligence + Healthcare’ in Zhejiang Province (2025–2027),” scored 7.48 in the PMC-Index, earning an “Excellent” rating and ranking fourth. This policy document, issued by the Zhejiang Provincial Health Commission, promotes the efficient application and high-quality development of artificial intelligence technology within the local healthcare sector. While indicators X3, X4, and X9 scored below average, the remaining six indicators achieved scores at or above the average level. Therefore, the recommended optimization path for indicators should be X4-X3-X9.

Policy P10, “Hubei Province Implementation Plan for Accelerating the Application of Artificial Intelligence in Healthcare (2025–2027),” scored 7.13 on the PMC-Index, earning an “Excellent” rating and ranking eighth. Indicators X3, X4, and X8 scored below average. This may stem from the policy’s failure to leverage Chongqing’s strengths in manufacturing and its role as a western healthcare hub to define specialized tracks. It also lacks clarification on “the responsible entity for aligning corporate and hospital clinical needs,” “funding allocation ratios for core technology breakthroughs,” and the division of responsibilities among departments. Consequently, the measures lack concrete implementation mechanisms, hindering goal realization. Therefore, it is recommended to optimize the indicator sequence to X4-X3-X8.

## 5 Discussion

By synthesizing the results of the PMC-Index analysis of 10 representative policies in China’s smart healthcare sector, we can explore the core findings of this study. From the perspective of overall policy effectiveness, the selected representative policies demonstrate high comprehensive benefits. Most policies play a positive role in advancing smart healthcare development, enhancing healthcare service quality, and promoting the rational allocation of medical resources, reflecting the foresight and effectiveness of government policy formulation (34). In China, significant policy differences exist across regions: Eastern regions prioritize the application and innovation of high-end technologies, attracting high-tech enterprises and talent to enhance international competitiveness; the central region balances technological application with equitable access, improving primary healthcare systems to narrow urban–rural disparities; the western region focuses on basic healthcare accessibility, integrating specialized medical resources with smart healthcare. These differences stem from regional economic development levels, technological foundations, and healthcare needs. Based on the above research findings, six policy optimization pathways are proposed.

(1) In terms of policy timeliness. It can be improved by establishing a three-tiered timeliness system encompassing long, medium, and short duration. Such a pathway can be supported by “Federal Health Information Technology Strategic Plan” in The United States. As a medium- and long-term planning policy for the field of smart healthcare, it is formulated and updated by the Office of the National Coordinator for Health IT (ONC). The strategy is continuously adjusted with the development of technology and changes in the healthcare environment to guide the direction of development in the field of health information technology (35). Overall, this dynamic adjustment mechanism can be established to ensure policy timing aligns with healthcare development trends and thus to implement adaptive governance.
(2) In terms of policy objectives, which should be shifted from qualitative descriptions to the combination of quantitative and qualitative forms. For example, the European Union’s “Artificial Intelligence Act” (36) categorizes AI in smart healthcare as a high-risk area, requiring the bias rate threshold of algorithm training data to be ≤5%, and providers to submit interpretability reports including SHAP value analysis. This approach avoids the generalization of objectives and enables policy objectives to be more targeted and operable.
(3) In terms of policy content. They should be contextualized with requiring the integration of regional intelligence levels to develop personalized rules, coordinating resource allocation balance, which can more effectively promote policy implementation and development (37). In regions with higher levels of intelligence, policies can focus on encouraging innovation and deepening application. For example, this practice can promote the integration of smart healthcare with cutting-edge technologies such as big data and cloud computing. In regions with lower levels of intelligence, policies should focus on infrastructure construction and talent cultivation, increasing investment in information technology equipment. This enables smart healthcare policies to be effectively implemented and promoted in different regions, thereby more comprehensively driving the development of smart healthcare.
(4) In terms of policy measures. Policy measures are operationalized through a cross-departmental platform and coordination mechanism led by the National Health Commission (38). This clarifies responsibilities among stakeholders, specifies implementation procedures, timelines, standards, and evaluation methods to ensure effective execution. Moreover, existing research suggests that cross-departmental cooperation is more likely to enhance administrative efficiency (39–41). By engaging in international collaborations and embracing global best practices for data sharing and AI development, Japan can not only align with international standards but also contribute its unique datasets to global research initiatives (42).
(5) In terms of policy tools, focus on diversify policy tools by optimizing the scope of mandatory tools, increasing their usage frequency, strengthening publicity and promotion, leveraging the leading and radiating role of demonstration pilots, encouraging collaboration and exchange in smart healthcare research and development, and exploring new pathways for government-enterprise synergy embedded in the value chain (43).
(6) In terms of policy evaluation. The dynamic evaluation of policies, along with the theory of policy implementation and feedback, can provide theoretical support for improving the implementation of medical service policies (44). For example, various types of data collected during the implementation of policies, such as electronic medical record systems and medical information platforms, can be used to adjust and optimize policies in a timely manner based on monitoring and feedback results, forming a closed loop of “evaluation-feedback-optimization.” At the same time, the role of assessment as a baton should be played in implementing a regular meeting mechanism (45), which can achieve sustainable and efficient operation of smart healthcare.

## 6 Conclusion

This study focuses on the analysis of 10 healthcare AI policies enacted between 2015 and 2025, constructing a PMC index evaluation model for quantitative analysis of policy texts. The research results indicate that the national attention to AI in smart healthcare development is increasing, with policy feasibility and synergy overall improving. However, there are aspects overlooked in the policy formulation process. The policy timeliness mostly focuses on short to medium-term effects, lacking guiding outlines and implementation standards with long-term impacts, particularly lacking funding support and legal safeguards. Moreover, there is a lack of comprehensive planning, hindering the rapid implementation of policies. In conclusion, it is necessary for all countries to participate extensively to create the atmosphere of international cooperation, as well as the supportive environment. Through conducting cross-regional benchmarking analysis, the development direction can be calibrated and practical shortcomings can be addressed. Meanwhile, the sharing and circulation of medical data should be promoted to facilitate the mutual learning and application of advanced medical technologies, experiences and the results of benchmarking analysis. In this process, the driving force of policy flow should not be underestimated. Governments need to incorporate cross-regional benchmarking analysis into the design and implementation of systems, rely on unified standards to weave the safety net for intelligent medical care, and effectively promote the standardized and balanced development of intelligent medical care.

From the theoretical perspective, the smart healthcare policy evaluation index system constructed in this study contributes a new theoretical framework to the field of AI in smart healthcare governance. It breaks through the limitations of traditional policy evaluation models, precisely focuses on the characteristics of smart healthcare, and provides clear directional guidance for subsequent related theoretical research. The study’s methodology and recommendations can offer a replicable template for evidence-based policy design in countries facing similar challenges. From the practical perspective, by analyzing ten representative policies, the study identifies optimization directions for different policy levels. National policies require enhanced tool diversity, local policies should refine timelines and application scenarios, and traditional Chinese medicine policies need improved cross-departmental coordination. For policymakers, this evaluation system can be used to formulate more targeted and operable smart healthcare policies, avoiding blindness and generalization of policies. This provides precise, targeted improvement recommendations for policy formulation. For medical enterprises and scientific research institutions, this system helps them better understand policy orientation and allocate resources reasonably. For the public, more scientific and reasonable smart healthcare policies will bring higher quality and safer medical services.

There are some shortcomings in this study. First, the policy texts selected are predominantly from 2015 to 2025, with a higher proportion of policies from developed regions and specific sectors in the sample. The insufficient coverage of policies from central and western regions and grassroots healthcare may affect the broad applicability of the evaluation results. Second, the evaluation indicators and influencing factors were constructed without fully accounting for external dynamic variables such as infrastructure, public acceptance, cross-regional coordination, and emergencies, limiting the comprehensive assessment of actual policy effectiveness. Future research will expand the sample scope to incorporate policy and implementation data from regions with diverse resource endowments and specific scenarios, enhancing the model’s scientific rigor. Concurrently, factors such as infrastructure, public perception, and emergencies will be integrated into the analytical framework. By incorporating international comparative studies, a more universally applicable and regionally tailored policy optimization model will be developed. This will enable the formulation of smart healthcare governance strategies and implementation pathways that better align with practical needs.

## Data Availability

The original contributions presented in the study are included in the article/supplementary material, further inquiries can be directed to the corresponding author.
